# Supplementary material for: The Evolution and Characterization of the RNA Interference Pathways in Lophotrochozoa
Source: Genome Biol Evol. 2024 May 7;16(5):evae098. doi: 10.1093/gbe/evae098 (PMC11114477; doi:10.1093/gbe/evae098)
Supplement: evae098_Supplementary_Data [file evae098_supplementary_data.zip › Supplementary_materials.zip!/selected_miRNAs/NoGe_selected_miRNAs/mirdeep_pdf_files/NMRB01001262.1_Notospermus_geniculatus_strain_Ushimado_scaffold1262,_whole_genome_shotgun_sequence_26853.pdf]

Provisional ID	:	NMRB01001262.1_Notospermus_geniculatus_strain_Ushimado_scaffold1262,_whole_genome_shotgun_sequence_26853
Score total	:	245011.5
Score for star read(s)	:	3.9
Score for read counts	:	245002.7
Score for mfe	:	1.9
Score for randfold	:	0
Score for cons. seed	:	3
Total read count	:	480574
Mature read count	:	471927
Loop read count	:	16
Star read count	:	8631

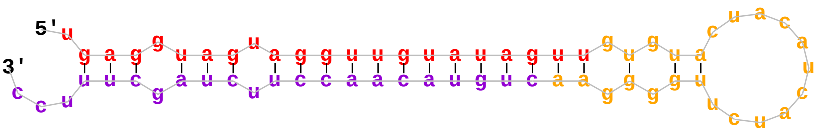

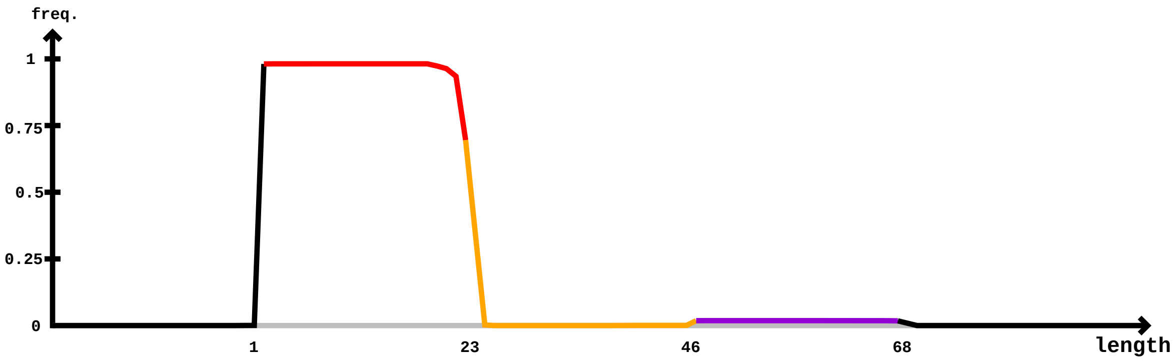

Mature **Star**

```
5'- caggagugggccccucagucugagguaguagguuguauaguuguguacuacaucaucuuggggaacuguacaaccuucuagcuuucccagagggcgccucaguacaacaucc   -3'    obs
    caggagugggccccucagucugagguaguagguuguauaguuguguacuacaucaucuuggggaacuguacaaccuucuagcuuucccagagggcgccucaguacaacaucc          exp
    ....(.((((((((((.....(((.(((.((((((((((((.(.((...........)).).)))))))))))).))).))).....))))).))).)).)...........   reads    mm    sample
    ..................ucugagguaguagguuguauagu.......................................................................   7        0     Mm2
    ...................cugagguaguagguuguauagu.......................................................................   1        0     Mm2
    ...................cugagguaguagguuguauaguu......................................................................   3        0     Mm2
    ...................cugagguaguaggGuguauaguu......................................................................   1        1     Mm2
    ....................ugagguaguagguuguUu..........................................................................   5        1     Mm2
    ....................ugagguaguagguuguau..........................................................................   78       0     Mm2
    ....................ugagguaguagguuguaA..........................................................................   6        1     Mm2
    ....................ugagguaguagguuguaG..........................................................................   1        1     Mm2
    ....................ugagguaguagguuguUua.........................................................................   1        1     Mm2
    ....................ugagguaguagguuguaua.........................................................................   93       0     Mm2
    ....................ugagguaguagguuguaAa.........................................................................   15       1     Mm2
    ....................ugagguaguGgguuguaua.........................................................................   1        1     Mm2
    ....................ugagguaguagguuguauU.........................................................................   81       1     Mm2
    ....................ugagguaguagguuguaCa.........................................................................   1        1     Mm2
    ....................ugagguaguaggAuguaua.........................................................................   1        1     Mm2
    ....................ugagguaguagCuuguauag........................................................................   1        1     Mm2
    ....................ugagCuaguagguuguauag........................................................................   1        1     Mm2
    ....................ugagguaguagguuguauaU........................................................................   10       1     Mm2
    ....................Ggagguaguagguuguauag........................................................................   2        1     Mm2
    ....................ugagguaguagguuguGuag........................................................................   1        1     Mm2
    ....................ugagguaguagguuguaCag........................................................................   5        1     Mm2
    ....................ugagguaguagguCguauag........................................................................   1        1     Mm2
    ....................ugagguUguagguuguauag........................................................................   1        1     Mm2
    ....................Agagguaguagguuguauag........................................................................   1        1     Mm2
    ....................ugagguaguagguuguCuag........................................................................   2        1     Mm2
    ....................ugagguaguagguuguaAag........................................................................   2        1     Mm2
    ....................ugagguaguagguuguaGag........................................................................   2        1     Mm2
    ....................ugCgguaguagguuguauag........................................................................   1        1     Mm2
    ....................ugagguaguagguuguauaA........................................................................   12       1     Mm2
    ....................ugagguaguaAguuguauag........................................................................   6        1     Mm2
    ....................ugagguaguagguuguauag........................................................................   695      0     Mm2
    ....................ugaggGaguagguuguauag........................................................................   1        1     Mm2
    ....................ugagguaguagguuguUuag........................................................................   3        1     Mm2
```

Mature Star

caggagugggccccucagucugagguaguagguuguauaguuguguacuacaucaucuuggggaacuguacaaccuucuagcuuucccagagggcgccucaguacaacaucc			
....................ugagguaguagguuguauagA...	24	1	Mm2
....................ugagguaguagguAguauagu...	2	1	Mm2
....................ugagguaguagguuguauagG...	5	1	Mm2
....................ugagguaguagguuAuauagu...	1	1	Mm2
....................ugagCuaguagguuguauagu...	1	1	Mm2
....................ugCgguaguagguuguauagu...	4	1	Mm2
....................ugagguaguaUguuguauagu...	8	1	Mm2
....................uUagguaguagguuguauagu...	4	1	Mm2
....................Agagguaguagguuguauagu...	1	1	Mm2
....................ugagguaguaAguuguauagu...	7	1	Mm2
....................ugagguaguagguugAauagu...	2	1	Mm2
....................ugagguaguaggCuguauagu...	1	1	Mm2
....................Cgagguaguagguuguauagu...	1	1	Mm2
....................ugagguUguagguuguauagu...	1	1	Mm2
....................ugagguaguagguCguauagu...	1	1	Mm2
....................ugagguaguagguuguaAagu...	21	1	Mm2
....................uAagguaguagguuguauagu...	1	1	Mm2
....................ugagguaguagguuguauUgu...	1	1	Mm2
....................ugagguaguagguugCauagu...	2	1	Mm2
....................ugagguaguagguuguauaUu...	1	1	Mm2
....................ugagguaguaCguuguauagu...	5	1	Mm2
....................ugGgguaguagguuguauagu...	10	1	Mm2
....................ugagguaguagguuguCuagu...	18	1	Mm2
....................ugagguaguagguuUuauagu...	2	1	Mm2
....................ugagguaguagguuguUuagu...	11	1	Mm2
....................Ngagguaguagguuguauagu...	3	1	Mm2
....................ugagguaguagguuguGuagu...	11	1	Mm2
....................ugagguaguagguuguauCgu...	1	1	Mm2
....................ugagguaUuagguuguauagu...	1	1	Mm2
....................ugagguaguagguuguauagC...	2	1	Mm2
....................ugagguaAuagguuguauagu...	1	1	Mm2
....................Ggagguaguagguuguauagu...	2	1	Mm2
....................ugagguaguagguuguaCagu...	39	1	Mm2
....................ugagguagGagguuguauagu...	2	1	Mm2
....................ugagguaguagguuguaGagu...	44	1	Mm2
....................ugagguaguUgguuguauagu...	1	1	Mm2
....................ugUgguaguagguuguauagu...	3	1	Mm2
....................ugagguaguGgguuguauagu...	1	1	Mm2
....................ugagguaguagguuguauaCu...	1	1	Mm2
....................ugagguaguagguuguauagu...	7052	0	Mm2
....................ugaggGaguagguuguauaguu..	2	1	Mm2
....................ugagguaguUgguuguauaguu..	6	1	Mm2
....................ugagguaguagguuguauUguu..	5	1	Mm2
....................ugagguCguagguuguauaguu..	2	1	Mm2
....................ugagguaUuagguuguauaguu..	1	1	Mm2
....................ugagguaguagguuguauaAuu..	3	1	Mm2
....................ugagguaguaUguuguauaguu..	22	1	Mm2
....................ugagguaguGgguuguauaguu..	11	1	Mm2
....................ugagguGguagguuguauaguu..	5	1	Mm2
....................ugagguaguagguuguauaguA..	147	1	Mm2
....................ugagguaguagguuguauagAu..	4	1	Mm2
....................ugagguUguagguuguauaguu..	7	1	Mm2
....................ugagguNguagguuguauaguu..	3	1	Mm2
....................ugagguaAuagguuguauaguu..	1	1	Mm2
....................ugagguaguagguuguauaguC..	2	1	Mm2
....................ugagguaguaggGuguauaguu..	2	1	Mm2
....................ugagguaguagCuuguauaguu..	2	1	Mm2
....................ugagguaguaCguuguauaguu..	23	1	Mm2
....................ugagguagCagguuguauaguu..	1	1	Mm2
....................ugagguaguagguuguaCaguu..	119	1	Mm2
....................ugaggCaguagguuguauaguu..	2	1	Mm2
....................ugagguaguagguuguauaguG..	16	1	Mm2
....................ugagguaguagguuguGuaguu..	28	1	Mm2
....................ugagguaguagguuguauGguu..	3	1	Mm2
....................ugUgguaguagguuguauaguu..	9	1	Mm2
....................Ggagguaguagguuguauaguu..	4	1	Mm2
....................ugagguaguagguuguauaCuu..	3	1	Mm2
....................ugagguaguaggCuguauaguu..	2	1	Mm2
....................ugagguaguCgguuguauaguu..	3	1	Mm2
....................ugagguagGagguuguauaguu..	1	1	Mm2

```
                    Mature                                     Star
caggagugggccccucagucugagguaguagguuguauaguuguguacuacaucaucuuggggaacuguacaaccuucuagcuuucccagagggcgccucaguacaacaucc
....................uAagguaguagguuguauaguu......................................................................    6       1    Mm2
....................ugagguaguagguuguUuaguu......................................................................    18      1    Mm2
....................ugagguaguaggAuguauaguu......................................................................    2       1    Mm2
....................ugaUguaguagguuguauaguu......................................................................    2       1    Mm2
....................ugagguagguAguauaguu.........................................................................    3       1    Mm2
....................ugagguagAagguuguauaguu......................................................................    1       1    Mm2
....................ugaggNaguagguuguauaguu......................................................................    1       1    Mm2
....................ugagguaguagguuguauagGu......................................................................    3       1    Mm2
....................ugCgguaguagguuguauaguu......................................................................    13      1    Mm2
....................ugagguaguaAguuguauaguu......................................................................    28      1    Mm2
....................ugagguaguagguuguCuaguu......................................................................    44      1    Mm2
....................ugagguaguagguCguauaguu......................................................................    2       1    Mm2
....................ugaAguaguagguuguauaguu......................................................................    4       1    Mm2
....................Cgagguaguagguuguauaguu......................................................................    7       1    Mm2
....................Ngagguaguagguuguauaguu......................................................................    7       1    Mm2
....................ugGgguaguagguuguauaguu......................................................................    25      1    Mm2
....................ugagguaguagguuUuauaguu......................................................................    1       1    Mm2
....................ugagguaguagguuguauaguu......................................................................    19324   0    Mm2
....................uCagguaguagguuguauaguu......................................................................    3       1    Mm2
....................ugagguaguagUuuguauaguu......................................................................    4       1    Mm2
....................ugagguaguagguuguauCguu......................................................................    1       1    Mm2
....................ugagguaguagguuguauaUuu......................................................................    2       1    Mm2
....................ugagguaguagguuguaAaguu......................................................................    89      1    Mm2
....................Agagguaguagguuguauaguu......................................................................    7       1    Mm2
....................ugagguaguagguuguaGaguu......................................................................    95      1    Mm2
....................ugagguaguagguuguauaguug.....................................................................    19      0    Mm2
....................ugagguaguagguuguauaguuA.....................................................................    297     1    Mm2
....................ugagguaguagguuguauaguAg.....................................................................    7       1    Mm2
....................ugagguaguagguuguauaguuC.....................................................................    14      1    Mm2
....................ugagguaguagguuguauaguuU.....................................................................    1843    1    Mm2
....................ugagguaguagguuguauaguuCu....................................................................    4       1    Mm2
....................ugagguaguagguuguauaguugu....................................................................    4       0    Mm2
....................ugagguaguagguuguauaguuUu....................................................................    88      1    Mm2
....................ugagguaguagguuguauaguuAu....................................................................    19      1    Mm2
....................ugagguaguagguuguauaguugA....................................................................    1       1    Mm2
....................ugagguaguagguuguauaguugug...................................................................    1       0    Mm2
....................ugagguaguagguuguauaguuguguac................................................................    1       0    Mm2
....................ugagguaguagguuguauaguuguguacu...............................................................    2       0    Mm2
.....................gagguaguagguuguauag........................................................................    1       0    Mm2
.....................gagguaguagguuguauaguu......................................................................    6       0    Mm2
.....................gagguaguagguuguauaguugA....................................................................    1       1    Mm2
.........................................uguguacuacaucaucuuggggaac..............................................    1       0    Mm2
..........................................guguacuacaucaucuugggg.................................................    1       0    Mm2
..........................................guguacuacaucaucuuggggа................................................    1       0    Mm2
..........................................guguacuacaucaucuuggggaa...............................................    1       0    Mm2
.................................................acaucaucuuggggaacuguacaaccuucuagcu.............................    1       0    Mm2
.................................................acaucaucuuggggaacuguacaaccuucuagcuuucc.........................    1       0    Mm2
......................................................aucuuggggaacuguacaaccuucuagcuuucc.........................    2       0    Mm2
......................................................aucuuggggaacuguacaaccuucuagcuuuccU........................    1       1    Mm2
.......................................................ucuuggggaacuguacaaccu....................................    1       0    Mm2
.......................................................ucuuggggaacuguacaaccuucuagcu.............................    1       0    Mm2
........................................................cuuggggaacuguacaaccuucuagcuuucc.........................    5       0    Mm2
.........................................................uuggggaacuguacaaccuucu.................................    6       0    Mm2
.........................................................uuggggaacuguacaaccuucuagcuuuc..........................    2       0    Mm2
.........................................................uuggggaacuguacaaccuucuagcuuucc.........................    10      0    Mm2
.........................................................uuggggaacuguacaaccuucuagcuuuccU........................    1       1    Mm2
..........................................................uggggaacuguacaaccuucuagcuuuc..........................    1       0    Mm2
..........................................................uggggaacuguacaaccuucuagcuuucc.........................    8       0    Mm2
...........................................................ggggaacuguacaaccuucu.................................    1       0    Mm2
...........................................................ggggaacuguacaaccuucuagcuuucc.........................    3       0    Mm2
...........................................................ggggaacuguUcaaccuucuagcuuucc.........................    1       1    Mm2
...........................................................ggggaacuguacaaccuucuagcuuuccU........................    1       1    Mm2
..............................................................gaacuguacaaccuucuagcuuucU.........................    1       1    Mm2
...............................................................aacuguacaaccuucuagcuuucc.........................    1       0    Mm2
................................................................acuguacaaccuucuagcuuucc.........................    1       0    Mm2
.................................................................cuguacaaccuucuagcu.............................    1       0    Mm2
.................................................................cuguacaaccuucuagcuuu...........................    1       0    Mm2
.................................................................cuguacaaccuucuagcuuuc..........................    35      0    Mm2
.................................................................cuguacaaccuucuagcuuUc..........................    1       1    Mm2
.................................................................cuguacaGccuucuagcuuucc.........................    1       1    Mm2
```

Mature Star

caggagugggccccucagucugagguaguagguuguauaguuguguacuacaucaucuuggggaacuguacaaccuucuagcuuucccagagggcgccucaguacaacaucc			
....cuguacaaccCucuagcuuucc.........................	1	1	Mm2
....cuguacaaccuucuagcuCucc.........................	1	1	Mm2
....cuguacaaccAucuagcuuucc.........................	1	1	Mm2
....cuguaUaaccuucuagcuuucc.........................	1	1	Mm2
....cuguacaaccuucuagcAuucc.........................	3	1	Mm2
....cuguacaaccuucuagcuuucU.........................	17	1	Mm2
....cuguacaaccuucuagcCuucc.........................	1	1	Mm2
....cuguacaaccuucuagcuuucc.........................	757	0	Mm2
....cugCacaaccuucuagcuuucc.........................	1	1	Mm2
....cuguacaaccuucuagAuuucc.........................	2	1	Mm2
....cuguacaaccGucuagcuuucc.........................	1	1	Mm2
....cAguacaaccuucuagcuuucc.........................	1	1	Mm2
....Auguacaaccuucuagcuuucc.........................	1	1	Mm2
....cuguacaaccuucuagUuuucc.........................	1	1	Mm2
....cuguacaaccuucuagcGuucc.........................	2	1	Mm2
....cuguacaaccuucuagcuuuccA........................	70	1	Mm2
....cuguacaaccuucuagcuuuccU........................	27	1	Mm2
....cuguacaaccuucuagcuuuccG........................	1	1	Mm2
....cuguacaaccuucuagcuuuccc........................	1	0	Mm2
....uguacaaccuucuagcuuucc.........................	14	0	Mm2
....uguacaaccuucuagcuuuccA........................	5	1	Mm2
....uguacaaccuucuagcuuuccU........................	5	1	Mm2
..................Ccugagguaguagguuguauaguu..	4	1	Mm1
...................cugagguaguagguuguauaguu..	14	0	Mm1
....................ugagguaguagguuguUu..	13	1	Mm1
....................ugagguaguagguuguaG..	1	1	Mm1
....................ugagguaguagguuguau..	137	0	Mm1
....................ugagguaguGgguuguau..	9	1	Mm1
....................ugCgguaguagguuguau..	1	1	Mm1
....................ugagAuaguagguuguau..	1	1	Mm1
....................ugagguaguagguuguCu..	1	1	Mm1
....................Ngagguaguagguuguau..	1	1	Mm1
....................ugagguaguagguuguaA..	13	1	Mm1
....................ugGgguaguagguuguau..	1	1	Mm1
....................ugagguaguagguuguaAa...	7	1	Mm1
....................ugagguaguagguuguaGa...	2	1	Mm1
....................ugagguaguagguuguaCa...	1	1	Mm1
....................ugagguaguagguuguaua...	99	0	Mm1
....................ugagguaguagguuguauU...	101	1	Mm1
....................ugagguaguaAguuguauag..	2	1	Mm1
....................ugagguaguagguuguauag..	837	0	Mm1
....................ugagguaguagguuguauaU..	27	1	Mm1
....................ugagguaguagguugCauag..	1	1	Mm1
....................ugagguaguaCguuguauag..	1	1	Mm1
....................ugagguaguagguuguCuag..	5	1	Mm1
....................Ngagguaguagguuguauag..	2	1	Mm1
....................ugGgguaguagguuguauag..	1	1	Mm1
....................ugagguaguagguuUuauag..	1	1	Mm1
....................ugagguaguagguuguaAag..	8	1	Mm1
....................ugagguUguagguuguauag..	1	1	Mm1
....................ugagguaguagguuguaCag..	7	1	Mm1
....................ugagguaguagguuguaGag..	5	1	Mm1
....................ugagguaguagguuguUuag..	2	1	Mm1
....................ugagguaguagguuguGuag..	1	1	Mm1
....................ugagguaguaUguuguauag..	1	1	Mm1
....................ugagguaguaCguuguauagu...	7	1	Mm1
....................ugagguaguagguuguauaUu...	2	1	Mm1
....................ugagguaguagguAguauagu...	2	1	Mm1
....................ugagguagCagguuguauagu...	4	1	Mm1
....................ugagguaguagguuguauGgu...	2	1	Mm1
....................uUagguaguagguuguauagu...	1	1	Mm1
....................ugagguNguagguuguauagu...	2	1	Mm1
....................ugagguaguaUguuguauagu...	7	1	Mm1
....................ugUgguaguagguuguauagu...	9	1	Mm1
....................ugagguaguagguuguauagA...	30	1	Mm1
....................ugagguaguagguuguGuagu...	7	1	Mm1
....................ugagguaguagUuuguauagu...	1	1	Mm1
....................ugagguaUuagguuguauagu...	11	1	Mm1
....................ugagguaguagguuguaAagu...	44	1	Mm1

```
                    Mature                                       Star
caggagugggccccucagucugagguaguagguuguauaguuguguacuacaucaucuuggggaacuguacaaccuucuagcuuucccagagggcgccucaguacaacaucc
....................ugagguaguagguuguaCagu.......................................................................   38     1   Mm1
....................ugagguGguagguuguauagu.......................................................................   1      1   Mm1
....................ugagguaguagguugAauagu.......................................................................   1      1   Mm1
....................Agagguaguagguuguauagu.......................................................................   1      1   Mm1
....................ugagguaguaAguuguauagu.......................................................................   10     1   Mm1
....................uAagguaguagguuguauagu.......................................................................   4      1   Mm1
....................ugagguaguagguuguauUgu.......................................................................   1      1   Mm1
....................ugaggNaguagguuguauagu.......................................................................   1      1   Mm1
....................Cgagguaguagguuguauagu.......................................................................   1      1   Mm1
....................ugagguaguagguuguCuagu.......................................................................   17     1   Mm1
....................ugaggCaguagguuguauagu.......................................................................   1      1   Mm1
....................Ngagguaguagguuguauagu.......................................................................   2      1   Mm1
....................ugaggAaguagguuguauagu.......................................................................   2      1   Mm1
....................ugagguaguagguuguaGagu.......................................................................   37     1   Mm1
....................ugagguaguagguuguUuagu.......................................................................   7      1   Mm1
....................ugagguaguagguCguauagu.......................................................................   2      1   Mm1
....................ugaUguaguagguuguauagu.......................................................................   1      1   Mm1
....................ugagguUguagguuguauagu.......................................................................   2      1   Mm1
....................ugCgguaguagguuguauagu.......................................................................   7      1   Mm1
....................ugagguaguagguuguauagG.......................................................................   1      1   Mm1
....................ugagguaguUgguuguauagu.......................................................................   1      1   Mm1
....................ugGgguaguagguuguauagu.......................................................................   7      1   Mm1
....................ugagguaguagguuguauagu.......................................................................   7077   0   Mm1
....................ugagguaguagAuuguauagu.......................................................................   6      1   Mm1
....................ugagguaguagguuguauaUuu......................................................................   1      1   Mm1
....................ugagguaguagAuuguauaguu......................................................................   1      1   Mm1
....................ugGgguaguagguuguauaguu......................................................................   38     1   Mm1
....................ugaAguaguagguuguauaguu......................................................................   1      1   Mm1
....................Ggagguaguagguuguauaguu......................................................................   1      1   Mm1
....................Ngagguaguagguuguauaguu......................................................................   14     1   Mm1
....................uAagguaguagguuguauaguu......................................................................   6      1   Mm1
....................ugagguaguagUuuguauaguu......................................................................   3      1   Mm1
....................ugagguaguagguuUuauaguu......................................................................   2      1   Mm1
....................ugagguagGagguuguauaguu......................................................................   1      1   Mm1
....................ugagguaguagguuguauGguu......................................................................   2      1   Mm1
....................ugagguaguagguuguauaAuu......................................................................   1      1   Mm1
....................ugagguaguaAguuguauaguu......................................................................   51     1   Mm1
....................ugagguaguagguuguUuaguu......................................................................   34     1   Mm1
....................ugagguagCagguuguauaguu......................................................................   1      1   Mm1
....................ugaggAaguagguuguauaguu......................................................................   3      1   Mm1
....................ugagguaguagguugAauaguu......................................................................   1      1   Mm1
....................ugagguaguagguuguaGaguu......................................................................   143    1   Mm1
....................ugagguaguUgguuguauaguu......................................................................   2      1   Mm1
....................ugagguGguagguuguauaguu......................................................................   9      1   Mm1
....................ugagguagAagguuguauaguu......................................................................   1      1   Mm1
....................ugagguaguagguuguauagCu......................................................................   1      1   Mm1
....................ugagguaguagguuguauUguu......................................................................   2      1   Mm1
....................ugagguaguaggAuguauaguu......................................................................   2      1   Mm1
....................ugagguaguagguuguCuaguu......................................................................   64     1   Mm1
....................ugagguaguaggGuguauaguu......................................................................   1      1   Mm1
....................ugagguaguagguAguauaguu......................................................................   2      1   Mm1
....................ugagguUguagguuguauaguu......................................................................   9      1   Mm1
....................ugagguaguagguuguauaguC......................................................................   1      1   Mm1
....................ugagguaguagguCguauaguu......................................................................   7      1   Mm1
....................ugagUuaguagguuguauaguu......................................................................   1      1   Mm1
....................uUagguaguagguuguauaguu......................................................................   12     1   Mm1
....................uNagguaguagguuguauaguu......................................................................   1      1   Mm1
....................ugagguaguagguuguauaguA......................................................................   241    1   Mm1
....................ugagAuaguagguuguauaguu......................................................................   3      1   Mm1
....................ugagguaguagguGguauaguu......................................................................   4      1   Mm1
....................ugagguaguaCguuguauaguu......................................................................   23     1   Mm1
....................ugagguaguagguuguGuaguu......................................................................   25     1   Mm1
....................ugaCguaguagguuguauaguu......................................................................   1      1   Mm1
....................ugagguaguagguuguauaguG......................................................................   4      1   Mm1
....................ugagguaguagguuguaCaguu......................................................................   167    1   Mm1
....................ugagguCguagguuguauaguu......................................................................   1      1   Mm1
....................ugCgguaguagguuguauaguu......................................................................   23     1   Mm1
....................ugagguaguagguuguauagAu......................................................................   4      1   Mm1
....................ugaggCaguagguuguauaguu......................................................................   5      1   Mm1
....................ugagguaguGgguuguauaguu......................................................................   5      1   Mm1
```

Mature **Star**

```
caggagugggccccucagucugagguaguagguuguauaguuguguacuacaucaucuuggggaacuguacaaccuucuagcuuucccagagggcgccucaguacaacaucc
....................ugaggGaguagguuguauaguu......................................................................  2      1  Mm1
....................Cgagguaguagguuguauaguu......................................................................  11     1  Mm1
....................ugagguaUuagguuguauaguu......................................................................  5      1  Mm1
....................ugaUguaguagguuguauaguu......................................................................  2      1  Mm1
....................ugagguaguagguuguauaguu......................................................................  27180  0  Mm1
....................ugUgguaguagguuguauaguu......................................................................  19     1  Mm1
....................ugagguaguagguuAuauaguu......................................................................  3      1  Mm1
....................ugagguaguCgguuguauaguu......................................................................  1      1  Mm1
....................ugagguaguagguuguauCguu......................................................................  3      1  Mm1
....................ugagguaguaUguuguauaguu......................................................................  27     1  Mm1
....................ugagguaguagguugCauaguu......................................................................  4      1  Mm1
....................Agagguaguagguuguauaguu......................................................................  9      1  Mm1
....................ugagguaguaggCuguauaguu......................................................................  3      1  Mm1
....................ugaggNaguagguuguauaguu......................................................................  1      1  Mm1
....................ugagguaguagguuguaAaguu......................................................................  131    1  Mm1
....................ugagCuaguagguuguauaguu......................................................................  2      1  Mm1
....................ugagguaguagguuguauaguuA.....................................................................  219    1  Mm1
....................ugagguaguagguuguauaguuU.....................................................................  2133   1  Mm1
....................ugagguaguUgguuguauaguug.....................................................................  1      1  Mm1
....................ugagguaguagguuguUuaguug.....................................................................  1      1  Mm1
....................ugagguaguagguuguauaguug.....................................................................  11     0  Mm1
....................ugagguaguagguuguauaguuUu....................................................................  59     1  Mm1
....................ugagguaguagguuguauaguuAu....................................................................  8      1  Mm1
....................ugagguaguagguuguauaguuguguaU................................................................  1      1  Mm1
.....................gagguaguagguuguauagu.......................................................................  19     0  Mm1
.....................gagguaguCgguuguauagu.......................................................................  1      1  Mm1
.....................gagguaguagguuguauaguu......................................................................  10     0  Mm1
.....................gagguaguagguuguauaguuU.....................................................................  1      1  Mm1
........................................................cuuggggaacuguacaaccuucuagcuuucc.........................  3      0  Mm1
..........................................................uggggaacuguacaaccuucuagcuuucc.........................  5      0  Mm1
............................................................gggaacuguacaaccuucuagc..............................  3      0  Mm1
................................................................cuguacaaccuucuagcu..............................  11     0  Mm1
................................................................cuguacaaccuucuagcuu.............................  7      0  Mm1
................................................................cuguacaaccuucuagAuuuc...........................  1      1  Mm1
................................................................cuguacaaccuucuagcuuuc...........................  43     0  Mm1
................................................................cuguacaaccuucuagcCuuc...........................  1      1  Mm1
................................................................cuguacaaccuucuagcAuuc...........................  1      1  Mm1
................................................................cuguacaaccuucuagcAuucc..........................  5      1  Mm1
................................................................Uuguacaaccuucuagcuuucc..........................  7      1  Mm1
................................................................cuguacaaccuucuagcGuucc..........................  7      1  Mm1
................................................................cuguacaaccuucuagcuuucc..........................  1099   0  Mm1
................................................................cuguaAaaccuucuagcuuucc..........................  1      1  Mm1
................................................................cuguacaaccuucuagAuuucc..........................  8      1  Mm1
................................................................cuguacaaccAucuagcuuucc..........................  1      1  Mm1
................................................................cuguacUaccuucuagcuuucc..........................  1      1  Mm1
................................................................cuguacaaccuucuagUuuucc..........................  2      1  Mm1
................................................................cuguaUaaccuucuagcuuucc..........................  1      1  Mm1
................................................................cuguacaaccuucuagcCuucc..........................  6      1  Mm1
................................................................Nuguacaaccuucuagcuuucc..........................  2      1  Mm1
................................................................cuguacaGccuucuagcuuucc..........................  1      1  Mm1
................................................................cuguacaaccGucuagcuuucc..........................  1      1  Mm1
................................................................cuguacaaccCucuagcuuucc..........................  2      1  Mm1
................................................................cuguacaaccuucuagcuuuccA.........................  32     1  Mm1
................................................................cuguacaaccuucuagcuuuccU.........................  45     1  Mm1

..................ucugagguaguagguuguauag........................................................................  21     0  Fm2
..................ucugagguaguagguuguauagu.......................................................................  87     0  Fm2
..................ucugagguaguagguuguauagG.......................................................................  1      1  Fm2
..................ucugagguGguagguuguauagu.......................................................................  1      1  Fm2
..................ucugagguaguagguugCauagu.......................................................................  1      1  Fm2
..................ucugagguaguagguuguauaguu......................................................................  2      0  Fm2
...................cugagguaguagguuguauaUu.......................................................................  1      1  Fm2
...................cugagguaguagguuguauagu.......................................................................  4      0  Fm2
...................cugagguaguagguuguauaguu......................................................................  10     0  Fm2
...................cugaggAaguagguuguauaguu......................................................................  2      1  Fm2
....................ugagguaguagguuguaA..........................................................................  3      1  Fm2
....................ugagguaguagguuguUu..........................................................................  9      1  Fm2
....................ugagguaguagguuguCu..........................................................................  1      1  Fm2
....................ugagguaguagguuguau..........................................................................  94     0  Fm2
....................ugagguaguagguuguGu..........................................................................  1      1  Fm2
```

Mature **Star**

```
caggagugggccccucagucugagguaguagguuguauaguuguguacuacaucaucuuggggaacuguacaaccuucuagcuuucccagagggcgccucaguacaacaucc
....................ugagguaguaCguuguaua.........................................................................   2      1   Fm2
....................ugagguaguagguuguaAa.........................................................................   13     1   Fm2
....................ugagguaguagguuguaGa.........................................................................   1      1   Fm2
....................ugagguaguagguuguUua.........................................................................   1      1   Fm2
....................ugagguaguagguuguaua.........................................................................   233    0   Fm2
....................ugagguaguagguuguaCa.........................................................................   2      1   Fm2
....................ugagguaguagguuguauU.........................................................................   130    1   Fm2
....................ugagguaguagguuguaGag........................................................................   5      1   Fm2
....................ugagguaguagguuguauaA........................................................................   4      1   Fm2
....................ugagguaguagguuguaCag........................................................................   6      1   Fm2
....................ugagguagGagguuguauag........................................................................   1      1   Fm2
....................ugagguaguaAguuguauag........................................................................   3      1   Fm2
....................ugagguaguaCguuguauag........................................................................   1      1   Fm2
....................Ngagguaguagguuguauag........................................................................   1      1   Fm2
....................Agagguaguagguuguauag........................................................................   2      1   Fm2
....................uUagguaguagguuguauag........................................................................   3      1   Fm2
....................ugagguaguagguuguauag........................................................................   956    0   Fm2
....................ugagguaguagguuguauaU........................................................................   10     1   Fm2
....................uAagguaguagguuguauag........................................................................   1      1   Fm2
....................ugagguaguagguugGauag........................................................................   1      1   Fm2
....................ugagguaguUgguuguauag........................................................................   1      1   Fm2
....................ugagguaguagguuguGuag........................................................................   1      1   Fm2
....................ugagguaguaUguuguauag........................................................................   1      1   Fm2
....................ugagguaguagguuguaAag........................................................................   3      1   Fm2
....................ugagguaguagguGguauag........................................................................   1      1   Fm2
....................ugagguaguagguuguCuag........................................................................   4      1   Fm2
....................ugagCuaguagguuguauagu.......................................................................   1      1   Fm2
....................ugagguaguaUguuguauagu.......................................................................   12     1   Fm2
....................ugagguaguagCuuguauagu.......................................................................   2      1   Fm2
....................ugaAguaguagguuguauagu.......................................................................   5      1   Fm2
....................ugagguNguagguuguauagu.......................................................................   1      1   Fm2
....................ugagUuaguagguuguauagu.......................................................................   1      1   Fm2
....................ugagguaguaggAuguauagu.......................................................................   2      1   Fm2
....................ugagguaguagguuguauUgu.......................................................................   2      1   Fm2
....................Cgagguaguagguuguauagu.......................................................................   3      1   Fm2
....................ugagguagCagguuguauagu.......................................................................   1      1   Fm2
....................ugagguaguagguuguGuagu.......................................................................   15     1   Fm2
....................ugagguaguaAguuguauagu.......................................................................   31     1   Fm2
....................ugagguaguagguuguUuagu.......................................................................   28     1   Fm2
....................ugagguaguagguuUuauagu.......................................................................   1      1   Fm2
....................Agagguaguagguuguauagu.......................................................................   1      1   Fm2
....................ugagguaguagguCguauagu.......................................................................   2      1   Fm2
....................ugagguaguagguuguauagA.......................................................................   51     1   Fm2
....................ugGgguaguagguuguauagu.......................................................................   14     1   Fm2
....................ugagguaguagguuguauagu.......................................................................   17065  0   Fm2
....................ugagguagAagguuguauagu.......................................................................   2      1   Fm2
....................ugCgguaguagguuguauagu.......................................................................   4      1   Fm2
....................Ngagguaguagguuguauagu.......................................................................   9      1   Fm2
....................ugagguaguagguuAuauagu.......................................................................   4      1   Fm2
....................ugagguaguagguuguaCagu.......................................................................   84     1   Fm2
....................ugagguaguUgguuguauagu.......................................................................   6      1   Fm2
....................ugagguaUuagguuguauagu.......................................................................   2      1   Fm2
....................ugagguaguagUuuguauagu.......................................................................   3      1   Fm2
....................ugagguaguagguuCuauagu.......................................................................   1      1   Fm2
....................ugaggGaguagguuguauagu.......................................................................   2      1   Fm2
....................ugagguUguagguuguauagu.......................................................................   4      1   Fm2
....................uAagguaguagguuguauagu.......................................................................   7      1   Fm2
....................ugagguaguagguuguaGagu.......................................................................   86     1   Fm2
....................ugagguaguagguuguauagC.......................................................................   6      1   Fm2
....................ugagAuaguagguuguauagu.......................................................................   2      1   Fm2
....................ugagguaguaCguuguauagu.......................................................................   15     1   Fm2
....................ugagguaguagguuguauagG.......................................................................   25     1   Fm2
....................ugagguaguagguuguCuagu.......................................................................   43     1   Fm2
....................ugaggAaguagguuguauagu.......................................................................   1      1   Fm2
....................ugagguaguagguuguauaAu.......................................................................   2      1   Fm2
....................ugagguagGagguuguauagu.......................................................................   5      1   Fm2
....................ugagguaguagguuguaAagu.......................................................................   69     1   Fm2
....................uCagguaguagguuguauagu.......................................................................   2      1   Fm2
....................uUagguaguagguuguauagu.......................................................................   2      1   Fm2
....................ugaUguaguagguuguauagu.......................................................................   1      1   Fm2
```

```
                    Mature                                        Star
caggagugggccccucagucugagguaguagguuguauaguuguguacuacaucaucuuggggaacuguacaaccuucuagcuuucccagagggcgccucaguacaacaucc
....................ugagguaguagguugAauagu.......................................................................  5      1  Fm2
....................Ggagguaguagguuguauagu.......................................................................  2      1  Fm2
....................ugagguaguagguuguauaUu.......................................................................  1      1  Fm2
....................ugagguaguagguAguauagu.......................................................................  5      1  Fm2
....................ugagguaguGgguuguauagu.......................................................................  5      1  Fm2
....................ugagguaguagguugCauagu.......................................................................  3      1  Fm2
....................ugagguGguagguuguauagu.......................................................................  4      1  Fm2
....................ugaCguaguagguuguauagu.......................................................................  1      1  Fm2
....................ugUgguaguagguuguauagu.......................................................................  9      1  Fm2
....................ugagguaguaggCuguauagu.......................................................................  3      1  Fm2
....................ugagguaguCgguuguauaguu......................................................................  2      1  Fm2
....................ugagguGguagguuguauaguu......................................................................  11     1  Fm2
....................ugagguaguagguuguUuaguu......................................................................  71     1  Fm2
....................ugaUguaguagguuguauaguu......................................................................  8      1  Fm2
....................ugagCuaguagguuguauaguu......................................................................  4      1  Fm2
....................uUagguaguagguuguauaguu......................................................................  10     1  Fm2
....................ugagguaguaggAuguauaguu......................................................................  1      1  Fm2
....................ugagguaguagguugGauaguu......................................................................  3      1  Fm2
....................ugagguaguagUuuguauaguu......................................................................  9      1  Fm2
....................uCagguaguagguuguauaguu......................................................................  5      1  Fm2
....................ugagguaguagguuUuauaguu......................................................................  5      1  Fm2
....................ugagguagGagguuguauaguu......................................................................  5      1  Fm2
....................ugagguaguagguuguauaguG......................................................................  70     1  Fm2
....................ugagguaguaggGuguauaguu......................................................................  2      1  Fm2
....................ugagguaguagguuguauUguu......................................................................  13     1  Fm2
....................ugagguaguagguuguauagGu......................................................................  6      1  Fm2
....................ugaggAaguagguuguauaguu......................................................................  12     1  Fm2
....................Ggagguaguagguuguauaguu......................................................................  6      1  Fm2
....................ugGgguaguagguuguauaguu......................................................................  75     1  Fm2
....................ugagguaguagCuuguauaguu......................................................................  1      1  Fm2
....................ugaCguaguagguuguauaguu......................................................................  2      1  Fm2
....................ugagguaguaUguuguauaguu......................................................................  54     1  Fm2
....................ugagguaguagguuguauaguu......................................................................  60568  0  Fm2
....................ugagguaguagguuguauaUuu......................................................................  8      1  Fm2
....................ugagguaguagguuguaAaguu......................................................................  257    1  Fm2
....................ugagAuaguagguuguauaguu......................................................................  8      1  Fm2
....................ugagguaguagguGguauaguu......................................................................  3      1  Fm2
....................ugagguaguagguuguauaCuu......................................................................  1      1  Fm2
....................ugUgguaguagguuguauaguu......................................................................  32     1  Fm2
....................ugagguaguagguCguauaguu......................................................................  23     1  Fm2
....................ugCgguaguagguuguauaguu......................................................................  32     1  Fm2
....................ugagguaguagguugAauaguu......................................................................  3      1  Fm2
....................ugagguaguUgguuguauaguu......................................................................  7      1  Fm2
....................ugaggGaguagguuguauaguu......................................................................  4      1  Fm2
....................ugagguaguagguugCauaguu......................................................................  4      1  Fm2
....................ugagguCguagguuguauaguu......................................................................  3      1  Fm2
....................ugagguaguagguuguaCaguu......................................................................  328    1  Fm2
....................ugagguNguagguuguauaguu......................................................................  7      1  Fm2
....................ugagguaguagguuguGuaguu......................................................................  74     1  Fm2
....................ugagguaguagguuguauaAuu......................................................................  1      1  Fm2
....................ugagguaguagguuguauagCu......................................................................  6      1  Fm2
....................Cgagguaguagguuguauaguu......................................................................  15     1  Fm2
....................ugagguaguagguuguauCguu......................................................................  3      1  Fm2
....................ugagguaguagguuguauaguA......................................................................  628    1  Fm2
....................ugagguaguaAguuguauaguu......................................................................  93     1  Fm2
....................ugagguaguagguuguCuaguu......................................................................  140    1  Fm2
....................ugagguaUuagguuguauaguu......................................................................  5      1  Fm2
....................ugagguaguagAuuguauaguu......................................................................  3      1  Fm2
....................ugagguUguagguuguauaguu......................................................................  20     1  Fm2
....................ugagguaguagguuguauaguC......................................................................  6      1  Fm2
....................ugagguaguagguAguauaguu......................................................................  18     1  Fm2
....................ugagguaAuagguuguauaguu......................................................................  4      1  Fm2
....................ugaggNaguagguuguauaguu......................................................................  8      1  Fm2
....................ugagguaguagguuguauGguu......................................................................  8      1  Fm2
....................Ngagguaguagguuguauaguu......................................................................  28     1  Fm2
....................ugagguaguGgguuguauaguu......................................................................  26     1  Fm2
....................ugaggCaguagguuguauaguu......................................................................  13     1  Fm2
....................ugagguaguagguuguaGaguu......................................................................  331    1  Fm2
....................ugagguaguagguuAuauaguu......................................................................  13     1  Fm2
....................ugagguagCagguuguauaguu......................................................................  8      1  Fm2
```

```
                    Mature                                       Star
caggagugggccccucagucugagguaguagguuguauaguuguguacuacaucaucuuggggaacuguacaaccuucuagcuuucccagagggcgccucaguacaacaucc
....................ugagguaguaggCuguauaguu......................................................................  12    1  Fm2
....................ugagguaguagguuguauagAu......................................................................  10    1  Fm2
....................ugaAguaguagguuguauaguu......................................................................  5     1  Fm2
....................ugagUuaguagguuguauaguu......................................................................  5     1  Fm2
....................ugagguagAagguuguauaguu......................................................................  17    1  Fm2
....................ugagguaguagguuCuauaguu......................................................................  2     1  Fm2
....................ugagguaguaCguuguauaguu......................................................................  54    1  Fm2
....................Agagguaguagguuguauaguu......................................................................  15    1  Fm2
....................uAagguaguagguuguauaguu......................................................................  21    1  Fm2
....................ugagguaguagguuguauaguug.....................................................................  68    0  Fm2
....................ugagguUguagguuguauaguug.....................................................................  1     1  Fm2
....................ugagguaguagguuguauaguGg.....................................................................  2     1  Fm2
....................ugagguaguagguuguauaguuU.....................................................................  4942  1  Fm2
....................ugagguaguagguuguaAaguug.....................................................................  2     1  Fm2
....................ugagguaguagguuguauaguAg.....................................................................  13    1  Fm2
....................ugagguaguagguuguauaguuC.....................................................................  119   1  Fm2
....................ugagguaguagguuguaCaguug.....................................................................  1     1  Fm2
....................ugagguaguagguuguauaguuA.....................................................................  609   1  Fm2
....................ugagguaguagguuguauaguuCu....................................................................  27    1  Fm2
....................ugagguaguagguuguauaguuAu....................................................................  24    1  Fm2
....................ugagguaguagguuguauaguuUu....................................................................  401   1  Fm2
....................ugagguaguagguuguauaguugu....................................................................  6     0  Fm2
.....................gagguaguagguuguauagu.......................................................................  2     0  Fm2
.....................gagguaguagguuguauaguu......................................................................  17    0  Fm2
.....................gagguaguagguuguauCguu......................................................................  1     1  Fm2
.....................Uagguaguagguuguauaguu......................................................................  3     1  Fm2
.....................gagguaguagguuguaCaguu......................................................................  1     1  Fm2
.....................gagguaguagguuguauaguug.....................................................................  9     0  Fm2
.......................................................ucuuggggaacuguacaaccuucuagcuuucc.........................  1     0  Fm2
........................................................cuuggggaacuguacaacc.....................................  5     0  Fm2
.........................................................uuggggaacuguacaaccuucu.................................  6     0  Fm2
.........................................................uuggggaacuguacaacUuucuagcuuucc.........................  1     1  Fm2
.........................................................uuggggaacuguacaaccuuAuagcuuucc.........................  1     1  Fm2
.........................................................uuggggaacuguacaaccuucuagcuuucc.........................  56    0  Fm2
.........................................................uuggggaacuguacaaUcuucuagcuuucc.........................  1     1  Fm2
.........................................................uuggggaacuguacaaccuucuagUuuucc.........................  1     1  Fm2
..........................................................uggggaacuguacaaccuucuagcuuucc.........................  13    0  Fm2
..........................................................uggggaacuguacaaccuucuagcGuucc.........................  1     1  Fm2
...........................................................ggggaacuguacaaccuucuag...............................  8     0  Fm2
.................................................................cuguacaaccuucuagcu.............................  3     0  Fm2
.................................................................cuguacaaccuucuagcuuuc..........................  29    0  Fm2
.................................................................cuguacaaccuucuagcCuucc.........................  8     1  Fm2
.................................................................Uuguacaaccuucuagcuuucc.........................  2     1  Fm2
.................................................................cuguacaUccuucuagcuuucc.........................  1     1  Fm2
.................................................................cuguacaaccCucuagcuuucc.........................  2     1  Fm2
.................................................................cuguacaaccuucuagcAuucc.........................  4     1  Fm2
.................................................................Nuguacaaccuucuagcuuucc.........................  3     1  Fm2
.................................................................cuguacaaccuucuagGuuucc.........................  1     1  Fm2
.................................................................cuguacaaccuucuagcuuucU.........................  2     1  Fm2
.................................................................cuguacaaccuucuagAuuucc.........................  8     1  Fm2
.................................................................cuguacaaccAucuagcuuucc.........................  2     1  Fm2
.................................................................cuguacaaccuucuagcuuucc.........................  1128  0  Fm2
.................................................................cAguacaaccuucuagcuuucc.........................  1     1  Fm2
.................................................................cuguacaaccuCcuagcuuucc.........................  1     1  Fm2
.................................................................cuguacUaccuucuagcuuucc.........................  1     1  Fm2
.................................................................cuguacaaccuucuagUuuucc.........................  4     1  Fm2
.................................................................cuguacaaccuucuagcGuucc.........................  9     1  Fm2
.................................................................cuguacaaccuucuagcuuuccU........................  29    1  Fm2
.................................................................cuguacaaccuucuagcuuuccA........................  27    1  Fm2
..................................................................uguacaaccuucuagcuuucc.........................  7     0  Fm2

.................ucugagguaguagguugGauagu........................................................................  1     1  Fm1
.................ucugagguaguagguuguauagu........................................................................  10    0  Fm1
.................ucugagguaguagguuguauaguu.......................................................................  4     0  Fm1
..................cugagguaguagguuguauagu........................................................................  1     0  Fm1
..................cugagguaguagguuguauaguu.......................................................................  6     0  Fm1
..................cugagguaguagguugAauaguu.......................................................................  1     1  Fm1
..................Augagguaguagguuguauaguu.......................................................................  1     1  Fm1
....................ugagguaguagguuguau..........................................................................  95    0  Fm1
....................ugagguaguagguuguaA..........................................................................  4     1  Fm1
```

```
                    Mature                                       Star
caggagugggccccucagucugagguaguagguuguauaguuguguacuacaucaucuuggggaacuguacaaccuucuagcuuucccagagggcgccucaguacaacaucc
....................ugUgguaguagguuguau..........................................................................   1      1   Fm1
....................ugagguaguaCguuguau..........................................................................   2      1   Fm1
....................ugagguaguagguuguUu..........................................................................   11     1   Fm1
....................ugagguaguagUuuguaua.........................................................................   1      1   Fm1
....................ugagguaguagguuguaua.........................................................................   98     0   Fm1
....................ugagguaguagguuguUua.........................................................................   2      1   Fm1
....................ugagguaguagguuguaCa.........................................................................   1      1   Fm1
....................ugagguaguagguuguaAa.........................................................................   4      1   Fm1
....................ugagguaguagguuguauU.........................................................................   131    1   Fm1
....................ugGgguaguagguuguauag........................................................................   1      1   Fm1
....................ugUgguaguagguuguauag........................................................................   2      1   Fm1
....................ugagguaguagguuguauaA........................................................................   7      1   Fm1
....................ugagguaguagguuguaAag........................................................................   3      1   Fm1
....................ugagguaguagguuguauag........................................................................   643    0   Fm1
....................ugagguaguagguuguaGag........................................................................   5      1   Fm1
....................ugaAguaguagguuguauag........................................................................   1      1   Fm1
....................ugagguaguagguuguauaU........................................................................   3      1   Fm1
....................ugagguaguagguuguaCag........................................................................   3      1   Fm1
....................ugagguaguagguuguauUg........................................................................   1      1   Fm1
....................ugagguaguagguuguUuag........................................................................   1      1   Fm1
....................ugagguagAagguuguauagu.......................................................................   3      1   Fm1
....................Cgagguaguagguuguauagu.......................................................................   1      1   Fm1
....................ugagguaguaUguuguauagu.......................................................................   10     1   Fm1
....................ugagguaguagguuguGuagu.......................................................................   12     1   Fm1
....................ugagguCguagguuguauagu.......................................................................   1      1   Fm1
....................ugagguaguagCuuguauagu.......................................................................   1      1   Fm1
....................ugaggAaguagguuguauagu.......................................................................   1      1   Fm1
....................ugagguaguagguuguauagG.......................................................................   13     1   Fm1
....................ugagAuaguagguuguauagu.......................................................................   1      1   Fm1
....................ugagguaguaggCuguauagu.......................................................................   1      1   Fm1
....................ugagguaguagguuguCuagu.......................................................................   24     1   Fm1
....................ugagguaguagguGguauagu.......................................................................   1      1   Fm1
....................Ggagguaguagguuguauagu.......................................................................   2      1   Fm1
....................ugUgguaguagguuguauagu.......................................................................   8      1   Fm1
....................ugagguaguaCguuguauagu.......................................................................   9      1   Fm1
....................ugagguaguCgguuguauagu.......................................................................   1      1   Fm1
....................ugagguaguagguuguUuagu.......................................................................   10     1   Fm1
....................uUagguaguagguuguauagu.......................................................................   1      1   Fm1
....................ugaggCaguagguuguauagu.......................................................................   2      1   Fm1
....................ugagguaguaAguuguauagu.......................................................................   23     1   Fm1
....................ugagguaguagguuguaGagu.......................................................................   65     1   Fm1
....................ugagguaguagguuguauagu.......................................................................   9531   0   Fm1
....................ugagguagCagguuguauagu.......................................................................   1      1   Fm1
....................ugagguaguGgguuguauagu.......................................................................   3      1   Fm1
....................ugagguNguagguuguauagu.......................................................................   1      1   Fm1
....................Ngagguaguagguuguauagu.......................................................................   8      1   Fm1
....................ugagguaguagguuguauaCu.......................................................................   1      1   Fm1
....................ugagguaguagguuguauUgu.......................................................................   2      1   Fm1
....................ugagguaguagguuUuauagu.......................................................................   3      1   Fm1
....................ugagguaguagguugAauagu.......................................................................   1      1   Fm1
....................ugCgguaguagguuguauagu.......................................................................   10     1   Fm1
....................ugagguagGagguuguauagu.......................................................................   2      1   Fm1
....................ugagguaguagguuguaCagu.......................................................................   74     1   Fm1
....................ugaUguaguagguuguauagu.......................................................................   4      1   Fm1
....................ugGgguaguagguuguauagu.......................................................................   13     1   Fm1
....................ugagguaguagguuguaAagu.......................................................................   28     1   Fm1
....................ugagguGguagguuguauagu.......................................................................   10     1   Fm1
....................Agagguaguagguuguauagu.......................................................................   7      1   Fm1
....................ugaAguaguagguuguauagu.......................................................................   1      1   Fm1
....................ugagguaguagAuuguauagu.......................................................................   1      1   Fm1
....................ugagguaguaggAuguauagu.......................................................................   1      1   Fm1
....................ugagguaguagguuguauGgu.......................................................................   1      1   Fm1
....................ugagguaguagguuguauagA.......................................................................   8      1   Fm1
....................uCagguaguagguuguauagu.......................................................................   1      1   Fm1
....................ugagguaguagguuguauagC.......................................................................   4      1   Fm1
....................uAagguaguagguuguauagu.......................................................................   2      1   Fm1
....................ugagguCguagguuguauaguu......................................................................   3      1   Fm1
....................ugagguaguagguAguauaguu......................................................................   6      1   Fm1
....................ugagguaguagguuguaCaguu......................................................................   159    1   Fm1
....................ugagguaguUgguuguauaguu......................................................................   8      1   Fm1
```

Mature **Star**

```
caggagugggccccucagucugagguaguagguuguauaguuguguacuacaucaucuuggggaacuguacaaccuucuagcuuucccagagggcgccucaguacaacaucc
....................ugagguGguagguuguauaguu......................................................................   13      1    Fm1
....................ugagguaguagguuUuauaguu......................................................................   10      1    Fm1
....................ugagguaguagguuguauGguu......................................................................   1       1    Fm1
....................ugagguaguagguuguUuaguu......................................................................   28      1    Fm1
....................ugagguagGagguuguauaguu......................................................................   4       1    Fm1
....................ugagguaguagguCguauaguu......................................................................   6       1    Fm1
....................ugagguaguagguuguauUguu......................................................................   9       1    Fm1
....................ugagguaguaAguuguauaguu......................................................................   50      1    Fm1
....................ugagUuaguagguuguauaguu......................................................................   2       1    Fm1
....................ugagguaguagguuguauaguC......................................................................   11      1    Fm1
....................ugagguaguagguuguGuaguu......................................................................   35      1    Fm1
....................Ngagguaguagguuguauaguu......................................................................   20      1    Fm1
....................ugaCguaguagguuguauaguu......................................................................   1       1    Fm1
....................ugagguaguagguuguauaUuu......................................................................   4       1    Fm1
....................uUagguaguagguuguauaguu......................................................................   8       1    Fm1
....................ugagguaguagguuguauaguu......................................................................   28805   0    Fm1
....................ugaggCaguagguuguauaguu......................................................................   4       1    Fm1
....................ugaAguaguagguuguauaguu......................................................................   3       1    Fm1
....................ugagguaUuagguuguauaguu......................................................................   7       1    Fm1
....................ugagguaguagguuguauagCu......................................................................   1       1    Fm1
....................uAagguaguagguuguauaguu......................................................................   8       1    Fm1
....................ugagCuaguagguuguauaguu......................................................................   1       1    Fm1
....................ugagguaguaggAuguauaguu......................................................................   2       1    Fm1
....................ugagguUguagguuguauaguu......................................................................   6       1    Fm1
....................ugUgguaguagguuguauaguu......................................................................   29      1    Fm1
....................ugaggAaguagguuguauaguu......................................................................   6       1    Fm1
....................ugagguaguagguuguauagAu......................................................................   3       1    Fm1
....................ugagguaguagguuguauaguA......................................................................   131     1    Fm1
....................ugagguaguagguugCauaguu......................................................................   3       1    Fm1
....................ugagAuaguagguuguauaguu......................................................................   1       1    Fm1
....................ugagguaguaUguuguauaguu......................................................................   21      1    Fm1
....................Cgagguaguagguuguauaguu......................................................................   16      1    Fm1
....................ugagguaguagguuguauaguG......................................................................   25      1    Fm1
....................ugagguaguaggCuguauaguu......................................................................   8       1    Fm1
....................ugCgguaguagguuguauaguu......................................................................   21      1    Fm1
....................ugagguaguagguuguaAaguu......................................................................   106     1    Fm1
....................Agagguaguagguuguauaguu......................................................................   8       1    Fm1
....................ugagguaguagguuguaGaguu......................................................................   155     1    Fm1
....................ugagguaguagCuuguauaguu......................................................................   1       1    Fm1
....................ugagguaguaCguuguauaguu......................................................................   32      1    Fm1
....................ugagguaguagguuguCuaguu......................................................................   67      1    Fm1
....................ugagguagCagguuguauaguu......................................................................   3       1    Fm1
....................uCagguaguagguuguauaguu......................................................................   2       1    Fm1
....................ugaggGaguagguuguauaguu......................................................................   2       1    Fm1
....................ugGgguaguagguuguauaguu......................................................................   37      1    Fm1
....................ugagguaCuagguuguauaguu......................................................................   2       1    Fm1
....................ugagguagAagguuguauaguu......................................................................   1       1    Fm1
....................ugagguaguagguuAuauaguu......................................................................   3       1    Fm1
....................ugagguaguagguuguauagGu......................................................................   1       1    Fm1
....................ugaggNaguagguuguauaguu......................................................................   4       1    Fm1
....................ugagguaguagguugAauaguu......................................................................   3       1    Fm1
....................ugagguaguCgguuguauaguu......................................................................   2       1    Fm1
....................Ggagguaguagguuguauaguu......................................................................   4       1    Fm1
....................ugagguNguagguuguauaguu......................................................................   1       1    Fm1
....................ugagguaguGgguuguauaguu......................................................................   25      1    Fm1
....................ugagguaguagguuguauaguuU.....................................................................   2582    1    Fm1
....................ugagguaguagguuguauaguuC.....................................................................   73      1    Fm1
....................ugagguaguagguuguauaguuA.....................................................................   382     1    Fm1
....................ugGgguaguagguuguauaguug.....................................................................   1       1    Fm1
....................ugagguaguagguuguauaguug.....................................................................   33      0    Fm1
....................ugagguaguagguuguauaguuCu....................................................................   6       1    Fm1
....................ugagguaguagguuguauaguuUu....................................................................   132     1    Fm1
....................ugagguaguagguuguauaguuAu....................................................................   44      1    Fm1
....................ugagguaguagguuguauaguuguguacuacU............................................................   1       1    Fm1
....................ugagguaguagguuguauaguuguguacuacaucaucuuU....................................................   2       1    Fm1
.....................gagguaguagguuguauagu.......................................................................   1       0    Fm1
.....................gagguaguCgguuguauaguu......................................................................   1       1    Fm1
.....................gagguaguagguuguauaguu......................................................................   12      0    Fm1
.....................Uagguaguagguuguauaguu......................................................................   1       1    Fm1
.....................gagguaguagguuguauaguuU.....................................................................   1       1    Fm1
```

Mature   Star

```
caggagugggccccucagucugagguaguagguuguauaguuguguacuacaucaucuuggggaacuguacaaccuucuagcuuucccagagggcgccucaguacaacaucc
.............................................uacuacaucaucuuggggaacugua..........................................    4     0    Fm1
.....................................................caucuuggggaacuguacaaccuucuagcuuuccU........................    1     1    Fm1
......................................................aucuuggggaacuguacaaccuucuagcuuucc.........................    2     0    Fm1
.......................................................ucuuggggaacuguacaacc.....................................    3     0    Fm1
.......................................................ucuuggggaacuguacaaccuucuagcuuucc.........................    3     0    Fm1
.......................................................ucuuggggaacuguacaaccuucuagcuuuccU........................    1     1    Fm1
........................................................cuuggggaacuguacaaccuucuagcu.............................    3     0    Fm1
........................................................cuuggggaacuguacaaccuucuagcuuucc.........................    3     0    Fm1
........................................................cuuggggaacuguacaaccuucuagcuuuccU........................    2     1    Fm1
........................................................cuuggggaacuguacaaccuucuagcuuuccA........................    3     1    Fm1
.........................................................uuggggaacuguacaacc.....................................    1     0    Fm1
.........................................................uuggggaacuguacaaccuucu.................................    18    0    Fm1
.........................................................uuggggaacuguacaacUuucuagcuuucc.........................    1     1    Fm1
.........................................................uuggggaacuguacaaccuucuagcuuucc.........................    20    0    Fm1
.........................................................uuAgggaacuguacaaccuucuagcuuucc.........................    1     1    Fm1
.........................................................uuggggaacuguacaaccuucuagcuuuccU........................    6     1    Fm1
..........................................................uggggaacuguacaaccuucuagcuuucc.........................    12    0    Fm1
..........................................................uggggaacuguacaaccuucuagcuuucU.........................    1     1    Fm1
..........................................................uggggaacuguacaaccuucuagcuuuccU........................    1     1    Fm1
..........................................................uggggaacuguacaaccuucuagcuuuccA........................    1     1    Fm1
...........................................................ggggaacuguacaaccuucuag...............................    9     0    Fm1
............................................................gggaacuguacaaccuucuagU..............................    8     1    Fm1
.................................................................cuguacaaccuucuagcu.............................    2     0    Fm1
.................................................................cuguacaaccuucuagcuu............................    2     0    Fm1
.................................................................cuguacaaccuucuagcuuu...........................    3     0    Fm1
.................................................................cuguacaaccuucuagcCuu...........................    1     1    Fm1
.................................................................cuguacaaccuucuagcuuuc..........................    158   0    Fm1
.................................................................cuguacaaccuucuagcuCuc..........................    1     1    Fm1
.................................................................cuguacaaccuucuagcAuuc..........................    1     1    Fm1
.................................................................cuguacaaccuucuUgcuuucc.........................    1     1    Fm1
.................................................................cuguacaaccuucuagcuuucA.........................    4     1    Fm1
.................................................................cuguacaaccuucuagcuuucc.........................    3132  0    Fm1
.................................................................cuguacaaUcuucuagcuuucc.........................    1     1    Fm1
.................................................................cuguacaaccuucuagAuuucc.........................    14    1    Fm1
.................................................................cugCacaaccuucuagcuuucc.........................    1     1    Fm1
.................................................................cuguacaaccuAcuagcuuucc.........................    1     1    Fm1
.................................................................cAguacaaccuucuagcuuucc.........................    2     1    Fm1
.................................................................cuguacaaccGucuagcuuucc.........................    7     1    Fm1
.................................................................cuguGcaaccuucuagcuuucc.........................    1     1    Fm1
.................................................................Auguacaaccuucuagcuuucc.........................    1     1    Fm1
.................................................................cuguacaaccAucuagcuuucc.........................    3     1    Fm1
.................................................................cuguacaacUuucuagcuuucc.........................    8     1    Fm1
.................................................................cuguacaaccuucuagcuuuUc.........................    1     1    Fm1
.................................................................cCguacaaccuucuagcuuucc.........................    1     1    Fm1
.................................................................cuguacaaccuucuagcCuucc.........................    16    1    Fm1
.................................................................cuguacaaccuucuagcAuucc.........................    20    1    Fm1
.................................................................Uuguacaaccuucuagcuuucc.........................    3     1    Fm1
.................................................................cuUuacaaccuucuagcuuucc.........................    1     1    Fm1
.................................................................cuguacaaccuucuagcGuucc.........................    20    1    Fm1
.................................................................cuguacaaccuuUuagcuuucc.........................    3     1    Fm1
.................................................................cuguacaaccuucuagcuuucU.........................    31    1    Fm1
.................................................................cuguacaaccuucuagGuuucc.........................    4     1    Fm1
.................................................................Nuguacaaccuucuagcuuucc.........................    1     1    Fm1
.................................................................cuguacaaccuucuagUuuucc.........................    8     1    Fm1
.................................................................cuguacaaccuucAagcuuucc.........................    1     1    Fm1
.................................................................cuguacaaccuucuagcuuuccG........................    4     1    Fm1
.................................................................cuguacaaccuucuagcuuuccU........................    78    1    Fm1
.................................................................cuguacaaccuucuagcuuuccA........................    168   1    Fm1
..................................................................uguacaaccuucuagcuuucc.........................    22    0    Fm1
..................................................................uguacaaccuucuagcuuuccU........................    6     1    Fm1
..................................................................uguacaaccuucuagcuuuccA........................    12    1    Fm1

..................ucugagguaguagguuguauag........................................................................    1     0    Mm3
..................ucugagguaguagguuguauagu.......................................................................    4     0    Mm3
..................ucugagguaguagguuguauaguu......................................................................    3     0    Mm3
...................cugagguaguagguuguauagu.......................................................................    2     0    Mm3
...................cugagguaguagguuguauaguu......................................................................    5     0    Mm3
...................cugagguaguagguuguauaguuU.....................................................................    1     1    Mm3
....................ugUgguaguagguuguau..........................................................................    1     1    Mm3
....................ugagguaguagguuguaG..........................................................................    3     1    Mm3
```

```
                    Mature                                          Star
caggagugggccccucagucugagguaguagguuguauaguuguguacuacaucaucuuggggaacuguacaaccuucuagcuuucccagagggcgccucaguacaacaucc
....................ugagguaguagguuguau..........................................................................    146    0    Mm3
....................ugGgguaguagguuguau..........................................................................    1      1    Mm3
....................ugagguaguagguuguUu..........................................................................    9      1    Mm3
....................ugagguaguagguuguaC..........................................................................    2      1    Mm3
....................ugagguaguaggAuguau..........................................................................    1      1    Mm3
....................uAagguaguagguuguau..........................................................................    1      1    Mm3
....................ugagguaguagguuguaA..........................................................................    7      1    Mm3
....................ugagguaguagguuguUua.........................................................................    2      1    Mm3
....................ugagguaguagguuguaAa.........................................................................    13     1    Mm3
....................ugagguaguagguuguauU.........................................................................    126    1    Mm3
....................ugagguaguagguuguaua.........................................................................    125    0    Mm3
....................ugagguaguaUguuguaua.........................................................................    1      1    Mm3
....................ugagguaguagguuguaGa.........................................................................    2      1    Mm3
....................uAagguaguagguuguauag........................................................................    1      1    Mm3
....................ugagguaguagguuguaAag........................................................................    2      1    Mm3
....................ugagguaguagguuguauag........................................................................    543    0    Mm3
....................ugagguaguGgguuguauag........................................................................    1      1    Mm3
....................uUagguaguagguuguauag........................................................................    1      1    Mm3
....................uCagguaguagguuguauag........................................................................    1      1    Mm3
....................ugGgguaguagguuguauag........................................................................    1      1    Mm3
....................ugagguaguagguuguauaU........................................................................    19     1    Mm3
....................ugagguaguagguuguauaA........................................................................    10     1    Mm3
....................ugUgguaguagguuguauag........................................................................    1      1    Mm3
....................ugagguaguaCguuguauag........................................................................    1      1    Mm3
....................Ngagguaguagguuguauag........................................................................    1      1    Mm3
....................ugagguaguagguuguaCag........................................................................    1      1    Mm3
....................ugagguaguagguuguUuag........................................................................    1      1    Mm3
....................ugagguaguagguuguaGag........................................................................    2      1    Mm3
....................ugagguaguGgguuguauagu.......................................................................    2      1    Mm3
....................ugGgguaguagguuguauagu.......................................................................    7      1    Mm3
....................Agagguaguagguuguauagu.......................................................................    3      1    Mm3
....................ugagguaguagguAguauagu.......................................................................    2      1    Mm3
....................ugagguaguaCguuguauagu.......................................................................    4      1    Mm3
....................ugagguaguagguuguUuagu.......................................................................    7      1    Mm3
....................ugagguagCagguuguauagu.......................................................................    1      1    Mm3
....................ugUgguaguagguuguauagu.......................................................................    5      1    Mm3
....................ugagguagGagguuguauagu.......................................................................    1      1    Mm3
....................ugagguaguagguuguCuagu.......................................................................    25     1    Mm3
....................ugagguGguagguuguauagu.......................................................................    3      1    Mm3
....................uUagguaguagguuguauagu.......................................................................    2      1    Mm3
....................ugagguaguaAguuguauagu.......................................................................    11     1    Mm3
....................uNagguaguagguuguauagu.......................................................................    1      1    Mm3
....................ugagguaguUgguuguauagu.......................................................................    3      1    Mm3
....................ugagguaUuagguuguauagu.......................................................................    1      1    Mm3
....................ugagguUguagguuguauagu.......................................................................    3      1    Mm3
....................ugagAuaguagguuguauagu.......................................................................    1      1    Mm3
....................ugagguaguagguCguauagu.......................................................................    1      1    Mm3
....................ugagguaguagguuguauaUu.......................................................................    2      1    Mm3
....................ugagguaguagguuguGuagu.......................................................................    8      1    Mm3
....................ugagguaguagguuguauagG.......................................................................    5      1    Mm3
....................ugagguaguagguuguauUgu.......................................................................    3      1    Mm3
....................Ggagguaguagguuguauagu.......................................................................    2      1    Mm3
....................ugagguaguagguuguauagC.......................................................................    5      1    Mm3
....................ugagguaguagguuguauGgu.......................................................................    1      1    Mm3
....................Cgagguaguagguuguauagu.......................................................................    1      1    Mm3
....................ugagguaAuagguuguauagu.......................................................................    1      1    Mm3
....................ugagguaguagguuguauagA.......................................................................    20     1    Mm3
....................ugagguaguagguugGauagu.......................................................................    1      1    Mm3
....................ugagguaguagguuguaAagu.......................................................................    25     1    Mm3
....................Ngagguaguagguuguauagu.......................................................................    2      1    Mm3
....................ugagguaguagguuguauaAu.......................................................................    2      1    Mm3
....................ugaUguaguagguuguauagu.......................................................................    1      1    Mm3
....................ugagguaguagguuguauagu.......................................................................    6423   0    Mm3
....................ugagguaguaUguuguauagu.......................................................................    8      1    Mm3
....................uAagguaguagguuguauagu.......................................................................    3      1    Mm3
....................ugaAguaguagguuguauagu.......................................................................    1      1    Mm3
....................ugagguaguaggCuguauagu.......................................................................    1      1    Mm3
....................ugagguaguagguuguaCagu.......................................................................    38     1    Mm3
....................ugagguaguagguGguauagu.......................................................................    1      1    Mm3
....................ugagguaguagguuguaGagu.......................................................................    29     1    Mm3
```

Mature Star

caggagugggccccucagucugagguaguagguuguauaguuguguacuacaucaucuuggggaacuguacaaccuucuagcuuucccagagggcgccucaguacaacaucc			
....................ugagguaguagguuguaGaguu..	75	1	Mm3
....................ugagguaguagguuAuauaguu..	1	1	Mm3
....................ugagguaguagguuguauaCuu..	1	1	Mm3
....................ugagguaguaggCuguauaguu..	1	1	Mm3
....................ugagguaguaggGuguauaguu..	2	1	Mm3
....................ugagguaguagguuguauagAu..	1	1	Mm3
....................ugagguaguagguuguauaguu..	14796	0	Mm3
....................ugagguaguagguAguauaguu..	1	1	Mm3
....................ugagguNguagguuguauaguu..	1	1	Mm3
....................ugagguaguagguuguauGguu..	2	1	Mm3
....................ugagguCguagguuguauaguu..	1	1	Mm3
....................ugagguaguaCguuguauaguu..	10	1	Mm3
....................ugagguaguagguCguauaguu..	2	1	Mm3
....................ugagguaguagguuguauaAuu..	1	1	Mm3
....................ugGgguaguagguuguauaguu..	29	1	Mm3
....................ugaggCaguagguuguauaguu..	3	1	Mm3
....................ugCgguaguagguuguauaguu..	15	1	Mm3
....................ugagUuaguagguuguauaguu..	2	1	Mm3
....................ugaCguaguagguuguauaguu..	1	1	Mm3
....................ugagguaguagguuguUuaguu..	18	1	Mm3
....................ugagguaguaUguuguauaguu..	13	1	Mm3
....................ugaggGaguagguuguauaguu..	1	1	Mm3
....................ugagguaguagguuguauCguu..	4	1	Mm3
....................ugagguaguagguuguaCaguu..	87	1	Mm3
....................ugagguaCuagguuguauaguu..	3	1	Mm3
....................ugaggNaguagguuguauaguu..	1	1	Mm3
....................ugaAguaguagguuguauaguu..	1	1	Mm3
....................ugagguaguagUuuguauaguu..	1	1	Mm3
....................ugagguaguagguuguaAaguu..	62	1	Mm3
....................ugagguagCagguuguauaguu..	2	1	Mm3
....................ugagguaguCgguuguauaguu..	2	1	Mm3
....................uUagguaguagguuguauaguu..	1	1	Mm3
....................ugagguaUuagguuguauaguu..	1	1	Mm3
....................ugagguaguGgguuguauaguu..	12	1	Mm3
....................uAagguaguagguuguauaguu..	8	1	Mm3
....................ugagguaguagguGguauaguu..	2	1	Mm3
....................ugagguagGagguuguauaguu..	2	1	Mm3
....................ugagguGguagguuguauaguu..	1	1	Mm3
....................ugagguaguaAguuguauaguu..	27	1	Mm3
....................ugagguUguagguuguauaguu..	3	1	Mm3
....................Agagguaguagguuguauaguu..	3	1	Mm3
....................Ggagguaguagguuguauaguu..	1	1	Mm3
....................ugagguaguagguuguCuaguu..	39	1	Mm3
....................uCagguaguagguuguauaguu..	1	1	Mm3
....................ugagguaguagguuguauaguC..	4	1	Mm3
....................ugaggAaguagguuguauaguu..	5	1	Mm3
....................ugagguaguagguUuauaguu..	1	1	Mm3
....................ugagguagAagguuguauaguu..	2	1	Mm3
....................ugagguaguagguuguauaguG..	13	1	Mm3
....................ugagguaguagguuguauUguu..	4	1	Mm3
....................Ngagguaguagguuguauaguu..	5	1	Mm3
....................ugagguaguagguuguGuaguu..	13	1	Mm3
....................ugagguaguagguuguauaguA..	115	1	Mm3
....................ugagguaguagguuCuauaguu..	1	1	Mm3
....................ugagguaguUgguuguauaguu..	5	1	Mm3
....................ugagguaguagguuguauagCu..	3	1	Mm3
....................ugUgguaguagguuguauaguu..	14	1	Mm3
....................ugagguaguagguugGauaguu..	1	1	Mm3
....................Cgagguaguagguuguauaguu..	7	1	Mm3
....................ugagguaguagguuguauaUuu..	2	1	Mm3
....................Agagguaguagguuguauaguug...	1	1	Mm3
....................ugagguaguagguuguauaguuC...	13	1	Mm3
....................ugagguaguagguuguauaguuU...	2816	1	Mm3
....................ugagguaguagguuguauaguuA...	268	1	Mm3
....................ugagguaguagguuguauaguAg...	4	1	Mm3
....................ugagguaguagguuguCuaguug...	1	1	Mm3
....................Ngagguaguagguuguauaguug...	1	1	Mm3
....................ugagguaguagguuguauaguug...	23	0	Mm3
....................ugagguaguaCguuguauaguug...	1	1	Mm3
....................ugagguaguagguuguauaguGg...	1	1	Mm3

```
                    Mature                                       Star
caggagugggccccucagucugagguaguagguuguauaguuguguacuacaucaucuuggggaacuguacaaccuucuagcuuucccagagggcgccucaguacaacaucc
....................ugagguaguagguuguauaguAgu....................................................................    1     1    Mm3
....................ugagguaguagguuguauaguuUu....................................................................    94    1    Mm3
....................ugagguaguagguuguauaguuAu....................................................................    22    1    Mm3
....................ugagguaguagguuguauaguuCu....................................................................    1     1    Mm3
....................ugagguaguagguuguauaguugugG..................................................................    1     1    Mm3
....................ugagguaguagguuguauaguugugu..................................................................    1     0    Mm3
....................ugagguaguagguuguaGaguuguguacu...............................................................    1     1    Mm3
.....................gagguaguagguuguauagu.......................................................................    3     0    Mm3
.....................gagguaguagguuguauaguu......................................................................    10    0    Mm3
.....................Uagguaguagguuguauaguu......................................................................    1     1    Mm3
.....................gagguaguagguuguauaguuU.....................................................................    2     1    Mm3
.....................gagguaguagguuguauaguuA.....................................................................    1     1    Mm3
......................agguaguagguuguauaguu......................................................................    1     0    Mm3
......................agguaguagguuguauaguuU.....................................................................    2     1    Mm3
......................agguaguagguuguauaguug.....................................................................    1     0    Mm3
.......................gguaguagguuguauagu.......................................................................    1     0    Mm3
.......................gguaguagguuguauaguuU.....................................................................    1     1    Mm3
.........................................uguguacuacaucaucuugggU.................................................    1     1    Mm3
..........................................guguacuacaucaucuugggg.................................................    1     0    Mm3
..........................................guguacuacaucaucuuggggaa...............................................    1     0    Mm3
................................................uacaucaucuuggggaacuguacaacc.....................................    2     0    Mm3
..................................................caucaucuuggggaacuguacaaccuuc..................................    1     0    Mm3
......................................................aucuuggggaacuguacaaccuucuagc..............................    1     0    Mm3
......................................................aucuuggggaacuguacaaccuucuagcuuucc.........................    4     0    Mm3
.......................................................ucuuggggaacuguacaaccuucuagU..............................    1     1    Mm3
.........................................................uuggggaacuguacaaccuucu.................................    1     0    Mm3
...........................................................ggggaacuguacaaccuucuagcuuucc.........................    2     0    Mm3
............................................................gggaacuguacaaccuucuag...............................    2     0    Mm3
............................................................gggaacuguacaaccuucuagc..............................    1     0    Mm3
...............................................................aacuguacaaccuucuagcuuucc.........................    1     0    Mm3
.................................................................cuguacaaccuucuagcC.............................    1     1    Mm3
.................................................................cuguacaaccuucuagcu.............................    3     0    Mm3
.................................................................cuguacaaccuucuagcuuu...........................    2     0    Mm3
.................................................................cuguacaaccuucuagcuuuc..........................    30    0    Mm3
.................................................................cuguacaaccuucuagGuuucc.........................    1     1    Mm3
.................................................................cuUuacaaccuucuagcuuucc.........................    1     1    Mm3
.................................................................cCguacaaccuucuagcuuucc.........................    1     1    Mm3
.................................................................cuguacaaccCucuagcuuucc.........................    1     1    Mm3
.................................................................Auguacaaccuucuagcuuucc.........................    1     1    Mm3
.................................................................cuguacaaccuucuagcuuucU.........................    10    1    Mm3
.................................................................cuguacaaccuucuagcuuucc.........................    1116  0    Mm3
.................................................................cuguacaaccuucuagUuuucc.........................    2     1    Mm3
.................................................................Uuguacaaccuucuagcuuucc.........................    4     1    Mm3
.................................................................cuguacaaccuucuagAuuucc.........................    9     1    Mm3
.................................................................cuguacaaccuucuagcAuucc.........................    9     1    Mm3
.................................................................cuguaUaaccuucuagcuuucc.........................    1     1    Mm3
.................................................................cuguacaaccuuUuagcuuucc.........................    2     1    Mm3
.................................................................cuguacaaccAucuagcuuucc.........................    1     1    Mm3
.................................................................cuguacaGccuucuagcuuucc.........................    1     1    Mm3
.................................................................cuguacaaccuucuagcGuucc.........................    6     1    Mm3
.................................................................cuguacaaccuucuagcCuucc.........................    3     1    Mm3
.................................................................cuguacaaccGucuagcuuucc.........................    1     1    Mm3
.................................................................cuguacaaccuucuagcuuuccA........................    48    1    Mm3
.................................................................cuguacaaccuucuagcuuuccU........................    32    1    Mm3
..................................................................uguacaaccuucuagcuuucc.........................    8     0    Mm3
..................................................................uguacaaccuucuagcuuuccG........................    1     1    Mm3
..................................................................uguacaaccuucuagcuuuccU........................    4     1    Mm3
..................................................................uguacaaccuucuagcuuuccA........................    3     1    Mm3

..................ucugagguaguagguuguauag........................................................................    4     0    Fm3
..................ucugagguaguagguuguauagu.......................................................................    12    0    Fm3
...................cugagguaguagguuguauagu.......................................................................    3     0    Fm3
...................cugagguaguagguuguauaguu......................................................................    11    0    Fm3
...................cugagguagCagguuguauaguu......................................................................    1     1    Fm3
...................cugagguaguGgguuguauaguu......................................................................    1     1    Fm3
....................ugagCuaguagguuguau..........................................................................    1     1    Fm3
....................Agagguaguagguuguau..........................................................................    1     1    Fm3
....................ugagguaguagguuguaC..........................................................................    23    1    Fm3
....................ugagguaguagguuguUu..........................................................................    96    1    Fm3
....................ugagguaguagguAguau..........................................................................    2     1    Fm3
```

```
                    Mature                                       Star
caggagugggccccucagucugagguaguagguuguauaguuguguacuacaucaucuuggggaacuguacaaccuucuagcuuucccagagggcgccucaguacaacaucc
....................ugGgguaguagguuguau..........................................................................    6       1    Fm3
....................ugagguUguagguuguau..........................................................................    1       1    Fm3
....................ugagguaguagguuguCu..........................................................................    6       1    Fm3
....................ugagguaguagguuguaA..........................................................................    134     1    Fm3
....................ugagguaguagguuguGu..........................................................................    5       1    Fm3
....................ugagguaguaAguuguau..........................................................................    4       1    Fm3
....................ugagguaguCgguuguau..........................................................................    1       1    Fm3
....................ugagguaguaUguuguau..........................................................................    6       1    Fm3
....................ugagguaguagguuguau..........................................................................    2865    0    Fm3
....................ugagguaguGgguuguau..........................................................................    1       1    Fm3
....................ugagguaAuagguuguau..........................................................................    1       1    Fm3
....................ugagguaguagguuguaG..........................................................................    8       1    Fm3
....................ugagguaguaggAuguau..........................................................................    1       1    Fm3
....................ugagguaguagCuuguau..........................................................................    2       1    Fm3
....................Cgagguaguagguuguau..........................................................................    1       1    Fm3
....................ugagguaguUgguuguaua.........................................................................    1       1    Fm3
....................Agagguaguagguuguaua.........................................................................    2       1    Fm3
....................ugagguaguaCguuguaua.........................................................................    1       1    Fm3
....................ugagguaguagguuAuaua.........................................................................    3       1    Fm3
....................ugagguaguagguuguaGa.........................................................................    18      1    Fm3
....................ugagguaguagguuguaCa.........................................................................    11      1    Fm3
....................ugagguaguagguuguauU.........................................................................    1945    1    Fm3
....................ugagguaguagguuguauC.........................................................................    1       1    Fm3
....................ugagguaguGgguuguaua.........................................................................    1       1    Fm3
....................ugGgguaguagguuguaua.........................................................................    1       1    Fm3
....................ugagguaguagguuguaAa.........................................................................    106     1    Fm3
....................Cgagguaguagguuguaua.........................................................................    1       1    Fm3
....................ugaggGaguagguuguaua.........................................................................    1       1    Fm3
....................ugagguaguagguuguGua.........................................................................    2       1    Fm3
....................ugagguaguaUguuguaua.........................................................................    3       1    Fm3
....................ugagguCguagguuguaua.........................................................................    1       1    Fm3
....................ugagguaguagguCguaua.........................................................................    1       1    Fm3
....................ugagguaguagguuguUua.........................................................................    1       1    Fm3
....................ugCgguaguagguuguaua.........................................................................    3       1    Fm3
....................ugagguaguagguuguaua.........................................................................    1441    0    Fm3
....................ugagguaguaAguuguaua.........................................................................    2       1    Fm3
....................ugagguaguaCguuguauag........................................................................    9       1    Fm3
....................uCagguaguagguuguauag........................................................................    1       1    Fm3
....................ugGgguaguagguuguauag........................................................................    14      1    Fm3
....................ugagguUguagguuguauag........................................................................    3       1    Fm3
....................ugagguagCagguuguauag........................................................................    4       1    Fm3
....................Ngagguaguagguuguauag........................................................................    1       1    Fm3
....................ugagguaguaUguuguauag........................................................................    9       1    Fm3
....................ugUgguaguagguuguauag........................................................................    7       1    Fm3
....................ugagguagAagguuguauag........................................................................    1       1    Fm3
....................uUagguaguagguuguauag........................................................................    3       1    Fm3
....................ugagguaguagguuguauag........................................................................    9248    0    Fm3
....................ugagguGguagguuguauag........................................................................    2       1    Fm3
....................ugagguaguagguuguCuag........................................................................    11      1    Fm3
....................ugagguaguaAguuguauag........................................................................    15      1    Fm3
....................ugagguaguagguuguaCag........................................................................    51      1    Fm3
....................Cgagguaguagguuguauag........................................................................    4       1    Fm3
....................ugagguaguagguuguauaA........................................................................    147     1    Fm3
....................Agagguaguagguuguauag........................................................................    3       1    Fm3
....................ugaggCaguagguuguauag........................................................................    2       1    Fm3
....................ugagguaguGgguuguauag........................................................................    5       1    Fm3
....................ugagguaguagguuguUuag........................................................................    11      1    Fm3
....................ugagguaguagguuguauUg........................................................................    1       1    Fm3
....................uAagguaguagguuguauag........................................................................    4       1    Fm3
....................ugagguaguagguAguauag........................................................................    2       1    Fm3
....................ugagguaguagguugAauag........................................................................    1       1    Fm3
....................ugagguaguagguuguaGag........................................................................    56      1    Fm3
....................ugaggGaguagguuguauag........................................................................    3       1    Fm3
....................ugagguaUuagguuguauag........................................................................    1       1    Fm3
....................ugCgguaguagguuguauag........................................................................    6       1    Fm3
....................ugagguaguagguuguauCg........................................................................    1       1    Fm3
....................ugagguaguagguuUuauag........................................................................    1       1    Fm3
....................ugagCuaguagguuguauag........................................................................    1       1    Fm3
....................ugagguaguagguugCauag........................................................................    2       1    Fm3
....................ugagguaguagguuguauGg........................................................................    1       1    Fm3
```

```
                    Mature                                      Star
caggagugggccccucagucugagguaguagguuguauaguuguguacuacaucaucuuggggaacuguacaaccuucuagcuuucccagagggcgccucaguacaacaucc
....................ugagguaguagguCguauag........................................................................ 6      1  Fm3
....................ugagguNguagguuguauag........................................................................ 1      1  Fm3
....................ugaggNaguagguuguauag........................................................................ 2      1  Fm3
....................ugagguaguagguuguGuag........................................................................ 10     1  Fm3
....................ugagguaguaggCuguauag........................................................................ 1      1  Fm3
....................ugagguaguagguGguauag........................................................................ 1      1  Fm3
....................ugaggAaguagguuguauag........................................................................ 2      1  Fm3
....................ugagguaguagguuguauaU........................................................................ 177    1  Fm3
....................ugagguaguagguuguaAag........................................................................ 46     1  Fm3
....................ugCgguaguagguuguauagu....................................................................... 37     1  Fm3
....................ugaUguaguagguuguauagu....................................................................... 8      1  Fm3
....................ugagguaguagguGguauagu....................................................................... 6      1  Fm3
....................ugagCuaguagguuguauagu....................................................................... 1      1  Fm3
....................ugaAguaguagguuguauagu....................................................................... 7      1  Fm3
....................ugagguaguagCuuguauagu....................................................................... 2      1  Fm3
....................ugagguaguagguuguauagC....................................................................... 9      1  Fm3
....................ugagguaguagguuguauCgu....................................................................... 4      1  Fm3
....................ugagguagAagguuguauagu....................................................................... 8      1  Fm3
....................ugagguGguagguuguauagu....................................................................... 9      1  Fm3
....................ugagAuaguagguuguauagu....................................................................... 3      1  Fm3
....................ugagguaguagguuguaCagu....................................................................... 404    1  Fm3
....................ugagguaguagguuUuauagu....................................................................... 4      1  Fm3
....................ugagguaguagguuAuauagu....................................................................... 4      1  Fm3
....................ugagguaguagguuguauUgu....................................................................... 13     1  Fm3
....................ugagguaguUgguuguauagu....................................................................... 8      1  Fm3
....................ugagguaguagguuguauaAu....................................................................... 14     1  Fm3
....................ugagguaguaggCuguauagu....................................................................... 20     1  Fm3
....................uUagguaguagguuguauagu....................................................................... 22     1  Fm3
....................ugagguaguagguuguauaUu....................................................................... 34     1  Fm3
....................ugagguUguagguuguauagu....................................................................... 23     1  Fm3
....................ugagguaguagguuguaGagu....................................................................... 363    1  Fm3
....................ugagguaguagguuguGuagu....................................................................... 61     1  Fm3
....................uCagguaguagguuguauagu....................................................................... 6      1  Fm3
....................ugagguaUuagguuguauagu....................................................................... 10     1  Fm3
....................ugagguaguagguuCuauagu....................................................................... 4      1  Fm3
....................ugaggCaguagguuguauagu....................................................................... 14     1  Fm3
....................ugagguNguagguuguauagu....................................................................... 1      1  Fm3
....................ugUgguaguagguuguauagu....................................................................... 42     1  Fm3
....................ugagguaguagguAguauagu....................................................................... 12     1  Fm3
....................ugagguaguaUguuguauagu....................................................................... 55     1  Fm3
....................ugagguaguagUuuguauagu....................................................................... 2      1  Fm3
....................ugagguaguagguugGauagu....................................................................... 1      1  Fm3
....................ugagguaguagAuuguauagu....................................................................... 2      1  Fm3
....................ugagguaguCgguuguauagu....................................................................... 2      1  Fm3
....................Ggagguaguagguuguauagu....................................................................... 6      1  Fm3
....................ugagguaCuagguuguauagu....................................................................... 2      1  Fm3
....................ugagguaAuagguuguauagu....................................................................... 1      1  Fm3
....................ugagguaguagguuguauagG....................................................................... 14     1  Fm3
....................ugagguagGagguuguauagu....................................................................... 5      1  Fm3
....................ugagguCguagguuguauagu....................................................................... 4      1  Fm3
....................ugagguaguagguuguauaCu....................................................................... 1      1  Fm3
....................uAagguaguagguuguauagu....................................................................... 37     1  Fm3
....................ugagguaguagguugAauagu....................................................................... 7      1  Fm3
....................ugagguaguagguugCauagu....................................................................... 5      1  Fm3
....................Cgagguaguagguuguauagu....................................................................... 17     1  Fm3
....................ugagguaguGgguuguauagu....................................................................... 14     1  Fm3
....................Ngagguaguagguuguauagu....................................................................... 27     1  Fm3
....................ugagguaguaggAuguauagu....................................................................... 5      1  Fm3
....................ugagguaguaAguuguauagu....................................................................... 98     1  Fm3
....................ugagguaguaCguuguauagu....................................................................... 48     1  Fm3
....................ugaCguaguagguuguauagu....................................................................... 8      1  Fm3
....................ugagguaguagguCguauagu....................................................................... 10     1  Fm3
....................ugaggGaguagguuguauagu....................................................................... 9      1  Fm3
....................ugagguaguagguuguCuagu....................................................................... 131    1  Fm3
....................Agagguaguagguuguauagu....................................................................... 17     1  Fm3
....................ugagguaguagguuguauGgu....................................................................... 3      1  Fm3
....................ugagguaguagguuguauagu....................................................................... 63418  0  Fm3
....................ugGgguaguagguuguauagu....................................................................... 67     1  Fm3
....................ugagguagCagguuguauagu....................................................................... 6      1  Fm3
....................ugagguaguagguuguauagA....................................................................... 440    1  Fm3
```

Mature Star

caggagugggccccucagucugagguaguagguuguauaguuguguacuacaucaucuuggggaacuguacaaccuucuagcuuucccagagggcgccucaguacaacaucc			
....................ugagguaguagguuguaAagu...	273	1	Fm3
....................ugaggNaguagguuguauagu...	12	1	Fm3
....................ugagguaguagguuguUuagu...	68	1	Fm3
....................ugaggAaguagguuguauagu...	10	1	Fm3
....................ugNgguaguagguuguauagu...	1	1	Fm3
....................ugagguagCagguuguauaguu..	25	1	Fm3
....................uAagguaguagguuguauaguu..	63	1	Fm3
....................ugagUuaguagguuguauaguu..	5	1	Fm3
....................ugagguaAuagguuguauaguu..	8	1	Fm3
....................ugagguaguagguuAuauaguu..	20	1	Fm3
....................ugagguaUuagguuguauaguu..	11	1	Fm3
....................ugagguagAagguuguauaguu..	19	1	Fm3
....................ugaAguaguagguuguauaguu..	18	1	Fm3
....................ugagguaguagguugGauaguu..	4	1	Fm3
....................ugagguaguagguuguauaguA..	687	1	Fm3
....................ugagguaguagguuguauaguG..	23	1	Fm3
....................ugagguaguagguuguauaUuu..	16	1	Fm3
....................ugGgguaguagguuguauaguu..	185	1	Fm3
....................Agagguaguagguuguauaguu..	37	1	Fm3
....................ugagguaguagguuguauGguu..	12	1	Fm3
....................uNagguaguagguuguauaguu..	1	1	Fm3
....................ugagguaguagguugCauaguu..	22	1	Fm3
....................ugagguaguagguuguauagCu..	9	1	Fm3
....................ugagguaguagguuguauCguu..	8	1	Fm3
....................ugaggCaguagguuguauaguu..	24	1	Fm3
....................ugagguaguagguuguauaCuu..	5	1	Fm3
....................ugagguaguagguuUuauaguu..	4	1	Fm3
....................ugagguaguaggGuguauaguu..	2	1	Fm3
....................ugagguaguagguuguauaguC..	16	1	Fm3
....................ugagguaguagguuguaGaguu..	789	1	Fm3
....................ugagCuaguagguuguauaguu..	10	1	Fm3
....................ugaggGaguagguuguauaguu..	12	1	Fm3
....................ugCgguaguagguuguauaguu..	104	1	Fm3
....................ugagguaguagguuguauagAu..	4	1	Fm3
....................ugagguaguagAuuguauaguu..	9	1	Fm3
....................ugagguaguagguuguGuaguu..	136	1	Fm3
....................ugagguaguagguuguauUguu..	15	1	Fm3
....................Ngagguaguagguuguauaguu..	80	1	Fm3
....................Cgagguaguagguuguauaguu..	58	1	Fm3
....................ugaCguaguagguuguauaguu..	9	1	Fm3
....................ugagguaguagguugAauaguu..	14	1	Fm3
....................ugagguGguagguuguauaguu..	42	1	Fm3
....................ugagguaguagCuuguauaguu..	7	1	Fm3
....................ugagguaguagguGguauaguu..	11	1	Fm3
....................ugagguaguCgguuguauaguu..	15	1	Fm3
....................ugagguaguagguAguauaguu..	27	1	Fm3
....................ugagguaguagguuguCuaguu..	331	1	Fm3
....................ugagguagGagguuguauaguu..	8	1	Fm3
....................ugagguaguagguuguaCaguu..	850	1	Fm3
....................ugagguaguagguuguaAaguu..	593	1	Fm3
....................ugagguaguUgguuguauaguu..	35	1	Fm3
....................ugagguaguagguuCuauaguu..	2	1	Fm3
....................ugagguaguagguuguauaguu..	140887	0	Fm3
....................ugagguUguagguuguauaguu..	60	1	Fm3
....................ugagguaguaggCuguauaguu..	35	1	Fm3
....................ugagguCguagguuguauaguu..	22	1	Fm3
....................ugaUguaguagguuguauaguu..	29	1	Fm3
....................ugagguaCuagguuguauaguu..	3	1	Fm3
....................ugagguaguagguCguauaguu..	44	1	Fm3
....................ugagguaguagguuguUuaguu..	146	1	Fm3
....................uUagguaguagguuguauaguu..	40	1	Fm3
....................ugagguaguagguuguauaAuu..	5	1	Fm3
....................ugagguaguGgguuguauaguu..	56	1	Fm3
....................ugaggNaguagguuguauaguu..	16	1	Fm3
....................ugagguNguagguuguauaguu..	5	1	Fm3
....................ugagAuaguagguuguauaguu..	15	1	Fm3
....................ugagguaguaUguuguauaguu..	152	1	Fm3
....................ugagguaguagUuuguauaguu..	7	1	Fm3
....................ugagguaguaCguuguauaguu..	132	1	Fm3
....................uCagguaguagguuguauaguu..	19	1	Fm3

Mature Star

caggagugggccccucagucugagguaguagguuguauaguuguguacuacaucaucuuggggaacuguacaaccuucuagcuuucccagagggcgccucaguacaacaucc			
....................Ggagguaguagguuguauaguu..	14	1	Fm3
....................ugaggAaguagguuguauaguu..	22	1	Fm3
....................ugagguaguagguuguauagGu..	2	1	Fm3
....................ugUgguaguagguuguauaguu..	98	1	Fm3
....................ugagguaguaAguuguauaguu..	230	1	Fm3
....................ugagguaguaggAuguauaguu..	9	1	Fm3
....................ugagguaguagguuguauaguuC...	46	1	Fm3
....................ugagguaguagguuAuauaguug...	1	1	Fm3
....................ugagguaguagguuguauaguuU...	11990	1	Fm3
....................ugagguaguagguuguauaguug...	96	0	Fm3
....................ugagguaguagguuguauaguAg...	5	1	Fm3
....................ugagguaguagguuguauaguuA...	898	1	Fm3
....................ugagguaguagguuguauaguuAu..	32	1	Fm3
....................ugagguaguagguuguauaguuUu..	74	1	Fm3
....................ugagguaguagguuguauaguugu..	23	0	Fm3
....................ugagguaguagguuguauaguuCu..	7	1	Fm3
....................ugagguaguagguuguauaguuguA...	1	1	Fm3
....................ugagguaguagguuguauaguugugu..	4	0	Fm3
....................ugagguaguagguuguauaguuguguac..	2	0	Fm3
....................ugagguaguagguuguauaguuguguacu...	3	0	Fm3
.....................gagguaguagguuguauU...	3	1	Fm3
.....................Uagguaguagguuguauag..	1	1	Fm3
.....................gagguaguagguuguauag..	5	0	Fm3
.....................gagguaguagguuguauagu...	21	0	Fm3
.....................gagguagAagguuguauagu...	1	1	Fm3
.....................gagguUguagguuguauagu...	1	1	Fm3
.....................gagguaguagguuguauaguu..	53	0	Fm3
.....................gagguaguagUuuguauaguu..	1	1	Fm3
.....................gagguaguagguuguCuaguu..	1	1	Fm3
.....................Nagguaguagguuguauaguu..	1	1	Fm3
.....................Uagguaguagguuguauaguu..	2	1	Fm3
.....................gagguaguagguuguauaguug...	1	0	Fm3
.....................gagguaguagguuguauaguuU...	6	1	Fm3
......................agguaguagguuguauaU..	1	1	Fm3
......................agguaguagguuguauaguu..	3	0	Fm3
......................Ugguaguagguuguauaguu..	3	1	Fm3
......................agguaguagguuguauaguuU...	1	1	Fm3
......................agguaguagguuguauaguuguU...	1	1	Fm3
.......................gguaguagguuguauaAuu..	1	1	Fm3
.......................gguaguagguuguauaguu..	11	0	Fm3
.........................uaguagguuguauaguuU...	1	1	Fm3
.........................uaguagguuguauaguuguguacu...	2	0	Fm3
....uguguacuacaucaucuuggga...	5	0	Fm3
....uacaucaucuuggggaacuguacaacc.....................................	2	0	Fm3
....ucuuggggaacuguacaaccuucuag...............................	1	0	Fm3
....uuggggaacuguacaaccuucu.................................	1	0	Fm3
....cuguacaaccuucuagcu.............................	3	0	Fm3
....cuguacaaccuucuagcuu............................	1	0	Fm3
....cuguacaaccuucuagcuuuc..........................	2	0	Fm3
....cuguacaaccuucuagcuuucU.........................	1	1	Fm3
....cuguacaaccuucuagcCuucc.........................	1	1	Fm3
....Uuguacaaccuucuagcuuucc.........................	1	1	Fm3
....cuguacaaccuucuagcuuucc.........................	46	0	Fm3
....cuguacaaccGucuagcuuucc.........................	1	1	Fm3
....cuguacaaccAucuagcuuucc.........................	1	1	Fm3
....cuguacaaccuucuagcuuuccA........................	11	1	Fm3
....cuguacaaccuucuagcuuuccU........................	3	1	Fm3
....uguacaaccuucuagcuuuccU........................	1	1	Fm3
